# The Price of Tumor Control: An Analysis of Rare Side Effects of Anti-CTLA-4 Therapy in Metastatic Melanoma from the Ipilimumab Network

**DOI:** 10.1371/journal.pone.0053745

**Published:** 2013-01-14

**Authors:** Caroline J. Voskens, Simone M. Goldinger, Carmen Loquai, Caroline Robert, Katharina C. Kaehler, Carola Berking, Tanja Bergmann, Clemens L. Bockmeyer, Thomas Eigentler, Michael Fluck, Claus Garbe, Ralf Gutzmer, Stephan Grabbe, Axel Hauschild, Rüdiger Hein, Gheorghe Hundorfean, Armin Justich, Ullrich Keller, Christina Klein, Christine Mateus, Peter Mohr, Sylvie Paetzold, Imke Satzger, Dirk Schadendorf, Marc Schlaeppi, Gerold Schuler, Beatrice Schuler-Thurner, Uwe Trefzer, Jens Ulrich, Julia Vaubel, Roger von Moos, Patrik Weder, Tabea Wilhelm, Daniela Göppner, Reinhard Dummer, Lucie M. Heinzerling

**Affiliations:** 1 Department of Dermatology, University Hospital Erlangen, Erlangen, Germany; 2 Department of Dermatology, University Hospital Zurich, Zurich, Switzerland; 3 Department of Dermatology, University Hospital Mainz, Mainz, Germany; 4 Institute Gustave Roussy, Department of Dermatology, Villejuif, France; 5 Department of Dermatology, University Hospital Kiel, Kiel, Germany; 6 Department of Dermatology and Allergology, University of Munich LMU, Munich, Germany; 7 Department of Internal Medicine (Gastroenterology, Endocrinology, and Pneumology), University Hospital Erlangen, Erlangen, Germany; 8 Department of Dermatology and Allergy/Department of Pathology, Skin Cancer Center Hannover/Hannover Medical School, Hannover, Germany; 9 Department of Dermatology, University Hospital Tübingen, Tübingen, Germany; 10 Department of Dermatology, Hospital Hornheide, Münster, Germany; 11 Department of Dermatology/III. Medical Department, Technische Universität München, München, Germany; 12 Department of Dermatology, University Hospital Graz, Graz, Austria; 13 Department of Dermatology, Elbe Kliniken Buxtehude, Buxtehude, Germany; 14 Department of Dermatology, University Hospital Frankfurt, Frankfurt, Germany; 15 Department of Dermatology, University Hospital Essen, Essen, Germany; 16 Department of Oncology/Hematology and Dermatology, Cantonal Hospital St. Gallen, St. Gallen, Switzerland; 17 Department of Dermatology, University Hospital Charité Berlin, Berlin, Germany; 18 Department of Dermatology, Hospital Quedlinburg, Quedlinburg, Germany; 19 Department of Dermatology, Cantonal Hospital Graubünden, Chur, Switzerland; 20 Department of Dermatology, University Hospital Magdeburg, Magdeburg, Germany; The University of Queensland, Australia

## Abstract

**Background:**

Ipilimumab, a cytotoxic T-lymphocyte antigen-4 (CTLA-4) blocking antibody, has been approved for the treatment of metastatic melanoma and induces adverse events (AE) in up to 64% of patients. Treatment algorithms for the management of common ipilimumab-induced AEs have lead to a reduction of morbidity, e.g. due to bowel perforations. However, the spectrum of less common AEs is expanding as ipilimumab is increasingly applied. Stringent recognition and management of AEs will reduce drug-induced morbidity and costs, and thus, positively impact the cost-benefit ratio of the drug. To facilitate timely identification and adequate management data on rare AEs were analyzed at 19 skin cancer centers.

**Methods and Findings:**

Patient files (n = 752) were screened for rare ipilimumab-associated AEs. A total of 120 AEs, some of which were life-threatening or even fatal, were reported and summarized by organ system describing the most instructive cases in detail. Previously unreported AEs like drug rash with eosinophilia and systemic symptoms (DRESS), granulomatous inflammation of the central nervous system, and aseptic meningitis, were documented. Obstacles included patientś delay in reporting symptoms and the differentiation of steroid-induced from ipilimumab-induced AEs under steroid treatment. Importantly, response rate was high in this patient population with tumor regression in 30.9% and a tumor control rate of 61.8% in stage IV melanoma patients despite the fact that some patients received only two of four recommended ipilimumab infusions. This suggests that ipilimumab-induced antitumor responses can have an early onset and that severe autoimmune reactions may reflect overtreatment.

**Conclusion:**

The wide spectrum of ipilimumab-induced AEs demands doctor and patient awareness to reduce morbidity and treatment costs and true ipilimumab success is dictated by both objective tumor responses and controlling severe side effects.

## Introduction

Ipilimumab has been shown to enhance pre-exisiting immune responses, including antitumor responses, by directly blocking cytotoxic T-lymphocyte antigen-4 (CTLA-4) mediated T cell inhibition [1], [2] and is now FDA and EMA approved as treatment modality in patients with metastatic melanoma. One treatment cycle consists of four infusions at approximately $30,000 each for a total of $120,000 drug costs per treated patient. In general, tumor responses are long-lasting [3], yet relatively limited with responses in only 10–15% of patients [4], [5]. However, its application is associated with immune-related adverse events (irAEs) in up to 64% of patients [6] and detailed treatment algorithms for the management of commonly reported side-effects are provided by the manufacturer. Since CTLA-4 is inducibly expressed on virtually all T cells, ipilimumab has the potential to induce irAEs in a wide variety of tissues and organs. Single cases of unpredictable, in part astonishing, and difficult to treat, life-threatening or even fatal side-effects, have been reported including cases of nephropathy [7], myopathy [8], sarcoidosis [9], Guillain-Barré syndrome [10], uveitis, and leucopenia [11].

Since ipilimumab is increasingly being applied, the medical community will be confronted with new ipilimumab-induced side effects. To limit ipilimumab-related morbidity, stringent identification and immediate treatment of side-effects is crucial. Therefore, we summarized rare and difficult-to-treat ipilimumab-induced side effects among 19 skin cancer centers. In addition, we address specific hurdles, which we feel are critical for the success of CTLA-4-based immunotherapy.

## Methods

### Ethics Statement

This retrospective study was approved by the local institutional review board of the Friedrich-Alexander-Universität Erlangen-Nürnberg. In addition, all clinical protocols (44 in total among 19 participating study centers) were reviewed and approved by the local institutional review boards of each participating center and were performed according to Good Clinical Practice (GCP) and the Helsinki Declaration. In agreement with the local institutional review board of the Friedrich-Alexander-Universität Erlangen-Nürnberg, no written consent was obtained from included patients since the study was conducted completely anonymous.

### Study Centers and Treatment Settings

Participating study centers screened patient files for ipilimumab-associated AEs and reported them on a template. Common AEs (e.g., rash, colitis) were excluded. Based upon the authors’ discretion, additional information was requested for the 15 most compelling and instructive cases. Study centers and treatment settings are summarized in Table S1.

## Results

A total of 752 melanoma patients were treated with ipilimumab at 19 skin cancer centers and 120 AEs were reported. These included fatigue, flu-like symptoms, rigor/chills, eosinophilia and rashes (38 patients), which were not further evaluated. A total of 88 rare AEs in 82 patients affecting skin (23 patients), endocrine system (14 patients), nervous system (11 patients), liver (11 patients), respiratory tract (8 patients), gastrointestinal tract (6 patients), pancreas (3 patients), sinuses (3 patients), renal system (2 patients), musculosceletal system (2 patients), heart (1 patient), eyes (1 patient), and upper extremities (1 patient) were observed. In addition, a systemic grade IV anaphylactoid reaction and a fatal case of tumor mass liquefication were reported.

### Skin

**Figure 1:**
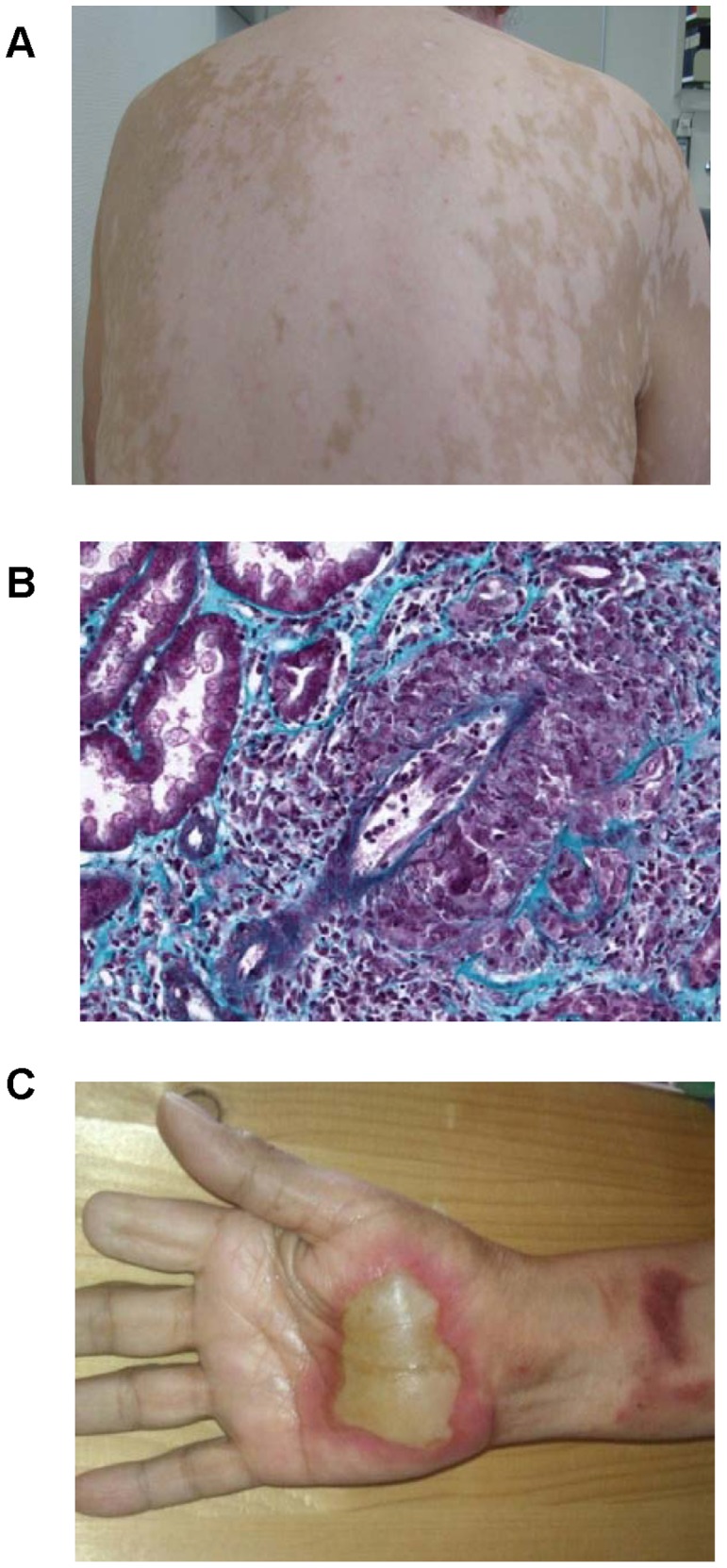
Ipilimumab-induced skin reactions and nephritis. Melanoma-associated hypopigmentation (MAH) in a patient exhibiting a partial clinical response (A). Masson’s trichome staining showed lymphocytic nephritis in a patient with an ipilimumab-induced drug rash with eosinophilia and systemic symptoms (DRESS) (B). Skin toxicity with the formation of blisters upon induction of treatment with ipilimumab in an area that had been radiated, five weeks earlier, in a patient with previous resection of the distal part of digit II due to an acrolentiginous melanoma (C).

Ipilimumab-induced skin reactions are common, yet rarely severe (1–4% grade 3/4 reactions). Maculopapular rashes occur in 10–50% of patients independently of dosage and pruritus has been documented in up to 29.6% [5]. Rarely, Sweets syndrome or Stevens-Johnson syndrome (SJS)/toxic epidermal necrolysis (TEN) have been observed (product monography). Importantly, melanoma-associated hypopigmentation (MAH) has been reported and postulated to be prognostically favorable [12]; [13]. In our study eight cases of MAH were reported (Figure 1A) and associated with one complete response (CR), one partial response (PR), one mixed response (MR), four stable diseases (SD) and one progressive disease (PD).

Other reported skin reactions included prurigo, acneiform rash, lichenoid exanthema, pyoderma gangraenosum-like ulcerations, skin toxicity in irradiated area, photosensitivity reaction and a drug rash with eosinophilia and systemic symptoms (DRESS) and detailed in Table 1 and Table 2 patient 6.

**Table 1:** Ipilimumab-induced cutaneous reactions.

side effect	onset (weeksafter start ofipilimumab)	treatment^a^ ofside effect	outcome ofside effect	age(years)	gender	primarytumor	stageb,c	previous systemictherapies	metastases*before*ipilimumab	remission*after*ipilimumab	clinicalresponse
drug rash with eosinophilia and systemic symptoms (DRESS)*	4	steroids (1 mg/kg)	resolved	77	M	skin	IV	none	lung, skin, LN	lung, skin, LN	PR
photosensitivity reaction*	2	none	resolved	47	F	skin	adjuvant	n/a	n/a	n/a	SD
skin toxicity in radiotherapy field*	1.5	none	resolved	59	F	skin	IV	none	lung, skin, adrenal gland,	none	SD
pyoderma gangraenosum-like ulcerations, diarrhea, cold clammy skin, fatigue	12	steroids (200 mg), antibiotics	permanent changes	57	F	skin	IV	IFN-α, DTIC, TKI (E7080)	liver, bone, spleen	none	PD
acneiform rash décolleté	12	local steroids	resolved	69	M	skin	IV	IFN-α	lung, liver, bone, soft tissue	none	SD
acneiform rash décolleté	14	local steroids	ongoing	50	M	skin	adjuvant	n/a	n/a	n/a	SD
acneiform rash décolleté	4	local steroids	resolved	38	M	skin	adjuvant	n/a	n/a	n/a	SD
lichenoid exanthema	12	local steroids	resolved	59	F	skin	IV	IFN-α	lung, skin	none	PD
prurigo	8	local steroids	resolved	64	M	skin	adjuvant	n/a	n/a	n/a	SD
prurigo and maculopapular exanthema	4	steroids, antihistamines	resolved	65	F	ocular	IV	DTIC	stomach, LN, mesenterial, lungs	none	PD
prurigo	11	local steroids	resolved	73	M	skin	IV	DTIC	soft tissue, LN, pericard, lung, bone	n/a	PD
prurigo	11	local steroids	resolved	72	F	skin	IV	IFN-α, DTIC, vaccination^d^	GIT, soft, tissue, skin	n/a	SD
prurigo	5	local steroids, antihistamines	resolved	81	M	skin	IV	DTIC, paclitaxel	GIT	n/a	PD
prurigo	6	local steroids	resolved	71	F	skin	IV	temozolomide	LN, brain	LN	MR
MAH	9	none	permanent changes	81	M	skin	IV	DTIC, paclitaxel	GIT	n/a	PD
MAH	14	local steroids	permanent changes	71	F	skin	IV	temozolomide	LN, brain	LN	MR
MAH	41	none	permanent changes	64	M	skin	adjuvant	n/a	n/a	n/a	SD
MAH	14	none	permanent changes	47	F	skin	adjuvant	n/a	n/a	n/a	SD
MAH	24	none	permanent changes	70	F	skin	adjuvant	n/a	n/a	n/a	SD
MAH	52	none	permanent changes	56	F	skin	IV	DTIC, sorafenib	lung	lung	CR
MAH	9	none	permanent changes	41	M	skin	IV	DTIC, gemcitabin+treosulfan	liver, suprarenal gland, LN, brain	n/a	SD
MAH and pruritic eczema	11	local steroids, antihistamines	permanent changes	65	M	skin	IV	DTIC	LN, skin, bone	LN, bone, skin	PR

* case is detailed in the result section.

a listed treatments are systemic treatments unless otherwise specified.

b tumor free high-risk stage III melanoma (AJCC 2009); adjuvant administration of ipilimumab.

c stage IV metastatic disease (AJCC 2009).

d MelanA-specific vaccination.

M indicates male; F, female; LN, lymph nodes; IFN-α, interferon-α; DTIC, dacarbazine; TKI, tyrosine kinase inhibitor; PR, partial response; SD, stable disease; PD, progressive disease; MR, mixed response; CR, complete response; MAH, melanoma-associated hypopigmentation.

**Table 2:** Ipilimumab-induced gastrointestinal, pancreatic and hepatic reactions.

side effect	onset (weeksafter start ofipilimumab)	treatment^a^ ofside effect	outcome ofside effect	age(years)	gender	primarytumor	stageb,c	previoussystemictherapies	metastases*before*ipilimumab	remission*after*ipilimumab	clinicalresponse
sigma perforation*	8	steroids (2 mg/kg), bowel resection	permanent changes	56	M	mucosal	IV	nilotinib, imatinib	lung, bone, LN	none	PD
colonic perforation*	7	steroids (2 mg/kg), infliximab (300 mg), surgery	permanent changes	74	F		IV	DTIC, vindesine+cisplatin, gemcitabine+treosulfan	lung, bone, soft tissue	lung, bone, soft tissue	PR
toxic megacolon*	16	colostomy	permanent changes	44	F	mucosal	IV	none	lung, LN	none	PD
small bowel perforation*	12	small bowel resection	permanent changes	67	M	skin	IV	IFN-α	skin, muscle, bone, retroperitoneal	skin, muscle, retroperitoneal	PR
ischaemic gastritis*	54	none	ongoing	72	F	skin	IV	n/a	intestinal, soft, tissue, skin	none	SD
diarrhea, maculo-papularexanthema, pruritus, fever,chills, dizziness	3	none	resolved	60	M	skin	IV	TVP	skin, brain, lung, soft tissue	brain	PR
pancreatitis	8	steroids	resolved	41	F	skin	adjuvant	n/a	n/a	n/a	SD
pancreatitis	12	steroids	resolved	68	F	skin	adjuvant	n/a	n/a	n/a	SD
fulminant hepatitis andcapillary leak, nephritis*	4	steroids	fatal	71	M	skin	IV	DTIC	LN, lung, liver	none	n/a
elevation of lipase/amylase	3	none	resolved	45	M	skin	IV	IFN-α	lung, skin, brain	none	PD
elevation of AST/ALT/GGT	9	steroids	resolved	73	F	skin	IV	IFN-α, TKI (RAF265), temozolomide	LN, bone	none	SD
elevation of AST/ALT	6	steroids	resolved	31	M	skin	adjuvant	n/a	n/a	n/a	SD
hepatitis	6	steroids	resolved	66	M	skin	adjuvant	n/a	n/a	n/a	SD
hepatitis	3	steroids	resolved	31	M	skin	adjuvant	n/a	n/a	n/a	SD
hepatitis	38	steroids	resolved	47	F	skin	adjuvant	n/a	n/a	n/a	SD
hepatitis	3	steroids	resolved	45	F	skin	adjuvant	n/a	n/a	n/a	SD
hepatitis	6	steroids, imurek	resolved	39	F	skin	adjuvant	n/a	n/a	n/a	SD
icterus	6	steroids, UV-therapy	resolved	66	M	skin	adjuvant	n/a	n/a	n/a	SD

* case is detailed in the result section.

a listed treatments are systemic treatments unless otherwise specified.

b tumor free high-risk stage III melanoma (AJCC 2009); adjuvant administration of ipilimumab.

c stage IV metastatic disease (AJCC 2009).

M indicates male; F, female; TVP, polychemotherapy with temozolomide+vinblastin+carboplatin; TKI, tyrosine kinase inhibitor RAF265; ALT, alanine transaminase; AST, aspartate transaminase; GGT, gamma-glutamyl transferase; LN, lymph nodes; IFN-α, interferon-α; DTIC, dacarbazine; PR, partial response; SD, stable disease; PD, progressive disease.

#### Patient 1– DRESS

A 77-year old metastatic melanoma patient was treated with ipilimumab (10 mg/kg body weight). The second ipilimumab infusion was combined with radiotherapy of the axillary region. Seven days after radiation, the patient presented with fever and overall performance deterioration. Two days thereafter, a diffuse maculo-papular rash without epidermal splits, necrosis, or mucosal symptoms developed, which rapidly progressed to erythrodermia without general symptoms. A hypereosinophilia at 2300/mm^3^ with normal hepatic function yet progressive renal failure (creatinine clearance 28 ml/min versus 84 ml/min at baseline) were observed one week after onset of the symptoms described above. A renal biopsy showed lymphocytic nephritis, indicative for a drug-related nephritis (Figure 1B). Oral prednisone (1 mg/kg body weight) was started and renal function, rash and hypereosinophilia normalised within one month. Importantly, staging showed a 40% tumor reduction. Overall, diagnosis of an ipilimumab-induced DRESS was likely due to the association of rash, hypereosinophilia, and renal failure at week four after initiation of therapy.

#### Patient 2–Skin toxicity in radiated area

After resection of an acrolentiginous melanoma a 59-year old patient developed metastases of the subcutaneous tissue of her right forearm, for which she was treated with surgery and radiotherapy. Additionally, lung and adrenal gland metastases appeared. Radiotherapy (20×2.5 Gray; total 50 Gray) was started three weeks before ipilimumab initiation while ipilimumab (3 mg/kg body weight) was started five days before the final radiation. Five days later the patient developed blisters within the radiated area (Figure 1C). These symptoms completely resolved under conservative local treatment with urea lotion and sulfadiazine silver and restaging showed stable disease. Importantly, an adverse reaction to the radiotherapy itself cannot be completely ruled out. However, the timely association with the initiation of ipilimumab therapy and the fact that no blister-formation was induced by radiotherapy alone, is highly suggestive for an ipilimumab-induced skin toxicity in the radiated area.

#### Patient 3 - Photosensitivity reaction

A 47-year old female patient received ipilimumab in an adjuvant setting (10 mg/kg body weight). Two weeks after the first infusion, erythematous maculae developed in sun-exposed regions a few hours after two short outdoor stays despite extensive sun protection (sun protection factor 50+; ultraviolet (UV)-B/UV-A). The erythema disappeared during the next five days. Further treatment was complicated by diarrhea (up to 10 times/day; treated with i.v. and subsequently oral steroids) 37 weeks after treatment initiation. In addition, circumscribed depigmented, non itchy areas below both knees, a pronounced itchy rash, erythematous macules and infiltrated plaques affecting the lumbar and inguinal region, the flexural areas, scalp, both palms and the lower right leg, were reported. Laboratory blood tests showed no signs of inflammation. Histological evaluation demonstrated a spongiotic epidermis with parakeratosis and acanthosis, a discrete edema in the dermal papillae and a perivascular infiltrate of lympho-histiocytes with some eosinophils. Based on these findings, a pruritic exanthema and/or a drug eruption was postulated, which completely subsided under treatment with topical steroids.

### Gastrointestinal Tract and Pancreas

Diarrhea/colitis is a common ipilimumab-induced AE [14], [15]. Severe colitis can result in bowel perforation or intractable bleeding requiring colectomy [4], [5], [16]–[18] and is associated with high mortality [19]. In our study, three bowel perforations, three cases of pancreatitis and one case of asymptomatic elevation of amylase and lipase (lipase 868 U/l; normal range 13–60 U/L, amylase 337 U/l; normal range <110 U/l) were reported (Table 2). In one patient, pancreatitis was preceded by an amenorrhea with hyperprolactemia (Table 3 patient 3).

**Table 3:** Ipilimumab-induced side effects of the endrocrine system.

side effect	onset (weeksafter start ofipilimumab)	treatment^a^ ofside effect	outcome ofside effect	age(years)	gender	primarytumor	stageb,c	previoussystemictherapies	metastases*before*ipilimumab	remission*after*ipilimumab	clinicalresponse
Hypophysitis*	7	steroids	permanentchanges	74	M	skin	IV	limb perfusion^d^	adrenal glands	none	SD
hypophysitis with symptoms ofbrain edema*	14	steroids	permanentchanges	M	67	skin	IV	IFN-α, cisplatin+vindesine+DTIC,DTIC+sorafenib,paclitaxel+carboplatin	skin, liver,lung, brain,LN	none	PD
amenorrhea, hyperprolactinemia, hypophysitis, with normal TSH/cortisol, pancreatitis	8	none	resolved	41	F	skin	adjuvant	n/a	n/a	n/a	SD
elevated TSH	24	none	ongoing	38	M	skin	adjuvant	n/a	n/a	n/a	SD
decreased TSH	12	steroids	resolved	68	F	skin	adjuvant	n/a	n/a	n/a	SD
generall hypophysal insufficiency (hypophysitis, hypothyreosis, hypogonadism)	15	steroids	ongoing	59	F	skin	IV	IFN-α, allovectin, DTIC, nilotinib	soft tissue, LN, lung	skin	PR
hypophysitis	10	steroids	resolved	57	M	skin	IV	temozolomide	brain	none	PD
hypophysitis	8	steroids, levothyroxine	resolved	56	F	skin	IV	DTIC	skin, peritoneal,	none	PD
hypophysitis	9	steroids	ongoing	60	F	mucosal	IV	IFN-α, DTIC, paclitaxel,docetaxel	parotis, LN, skin, lung	none	PD
hypophysitis	10	steroids, levothyroxin	permanent changes	31	M	skin	IV	DTIC, MEK-inhibitor	LN, skin	LN, skin	PR
hypophysitis	12	steroids, levothyroxine, testosterone	ongoing	72	M	skin	IV	DTIC, limb perfusion^d^	LN, spleen, lung, liver, muscle, skin	none	PD
hypophysitis+hepatitis	12	steroids	permanent changes	M	54	skin	adjuvant	n/a	n/a	n/a	SD
hypophysitis, pronounced fatigue, flu like symptoms	23	steroids	resolved	M	64	skin	adjuvant	n/a	n/a	n/a	SD
hypophysitis (hyperprolaktinaemia, low IGF-1, hyponatraemia), hypokaliaemia, hypophosphataemia	11	steroids	resolved	F	49	skin	IV	bevacizumab, temozolomide, DTIC, eldesine, platinol, paclitaxel, sorafenib	lung, liver, soft tissue, pancreas, LN, bone, skin, GIT	liver, LN	MR

* case is detailed in the result section.

a listed treatments are systemic treatments unless otherwise specified.

b tumor-free high-risk stage III melanoma (AJCC 2009); adjuvant administration of ipilimumab.

c stage IV metastatic disease (AJCC 2009).

d limb perfusion with melphalan.

M indicates male; F, female; LN, lymph nodes; IFN-α, interferon-α; DTIC, dacarbazine; GIT, gastrointestinal tract; IGF-1, insulin-like growth factor-1; TSH, thyroid-stimulating hormone; PR, partial response; SD, stable disease; PD, progressive disease; MR, mixed response.

#### Patient 4 - Sigma perforation

A 55-year old man with lung, bone and lymph node melanoma metastases and a history of diverticular disease developed diarrhea (9–10 times/day) and cramp-like abdominal pain two weeks after the third ipilimumab infusion (3 mg/kg body weight). Intravenous steroids were given and symptoms improved. Since the abdomen was soft without signs of rebound tenderness, steroids were continued orally (2 mg/kg body weight). However, after switching to oral steroids, acute abdominal pain with signs of peritonitis developed. Computed tomography (CT) imaging demonstrated pneumoperitoneum highly suspicious for a perforation. The perforated sigmoid was resected and a colostomy was performed. Histological findings demonstrated an exacerbated purulent diverticulitis (positive for *Prevotella intermedia*, streptococci and *Escherichia coli*) and perforation. Steroids were continued after surgery and subsequently tapered.

In this case the colitis initially seemed completely controlled by intravenous (i.v.) steroid treatment, yet rapidly deteriorated under steroid reduction. This implicates incomplete suppression of ipilimumab-triggered autoimmune effects or masking of symptoms under steroid treatment. Importantly, a known inflammatory condition like diverticular disease might represent a relative contraindication for ipilimumab. This may require special caution with prior ultrasound examination and/or prophylactic steroid treatment.

#### Patient 5 - Colonic perforation

A 74-year old melanoma patient with progression of disease despite prior polychemo- and radiotherapy showed a partial remission six weeks after initiation of ipilimumab treatment (3 mg/kg body weight). Five days later she reported diarrhea (10 times/day) that had been ongoing for five days. Treatment with i.v. prednisolone (2 mg/kg body weight) and loperamide (2 mg after each defecation) was initiated. Symptoms subsided within five days and she was continued on oral steroids (1 mg/kg body weight). After three days of oral steroids diarrhea recurred and i.v. prednisolone treatment (2 mg/kg body weight) was reinitiated. However, symptoms now were steroid-refractory and despite additional therapy with infliximab (300 mg absolute i.v.) an acute abdomen developed. A hemicolectomy with colostomy was performed due to perforation of the colon. Treatment with infliximab was continued every 6 weeks with final amelioration of the colitis.

#### Patient 6 - Toxic megacolon

A 44-year old woman with stage IV melanoma developed diarrhea (up to 30 times/day) and a subsequent acute abdomen sixteen weeks after initiation of ipilimumab treatment. X-ray showed ballooned bowels. A perforation was feared and a colostomy was performed to relief pressure. Interestingly, the colostomy showed no signs of healing and a revision was performed two weeks later. Histological findings showed ulcers and a granulocytic infiltrate in the mucosa. No bacterial or viral pathogens were found and the patient fully recovered from this AE.

#### Patient 7 - Small bowel perforation

Ipilimumab treatment (3 mg/kg body weight) was initiated in a 67-year old male with multiple melanoma metastases. Since the patient developed abdominal pain a colonoscopy was conducted but showed no signs of ulceration or colitis. However, the small intestine could not be investigated. Twelve weeks after the first ipilimumab treatment, the patient was admitted with acute abdomen and an emergency small bowel resection was performed. At this time, he also suffered from purulent peritonitis, thus no steroids were given. Staging showed a PR with regression of all metastases except bone lesions.

Detailed treatment algorithms for the management of ipilimumab-induced diarrhea/colitis exist [20]. Whereas in previous reports three out of four patients with colonic perforation were refractory to initial treatment with high-dose steroids [21], perforations in our study occurred after initial symptom improvement and steroid reduction. Steroids should be slowly tapered (30–60 days) and in cases of symptom recurrence, steroids should immediately be administered i.v. and if symptoms do not improve within 24 hours of therapy, additional immunosuppressive therapy (e.g. infliximab) should be initiated.

#### Patient 8 - Ischemic gastritis

**Figure 2:**
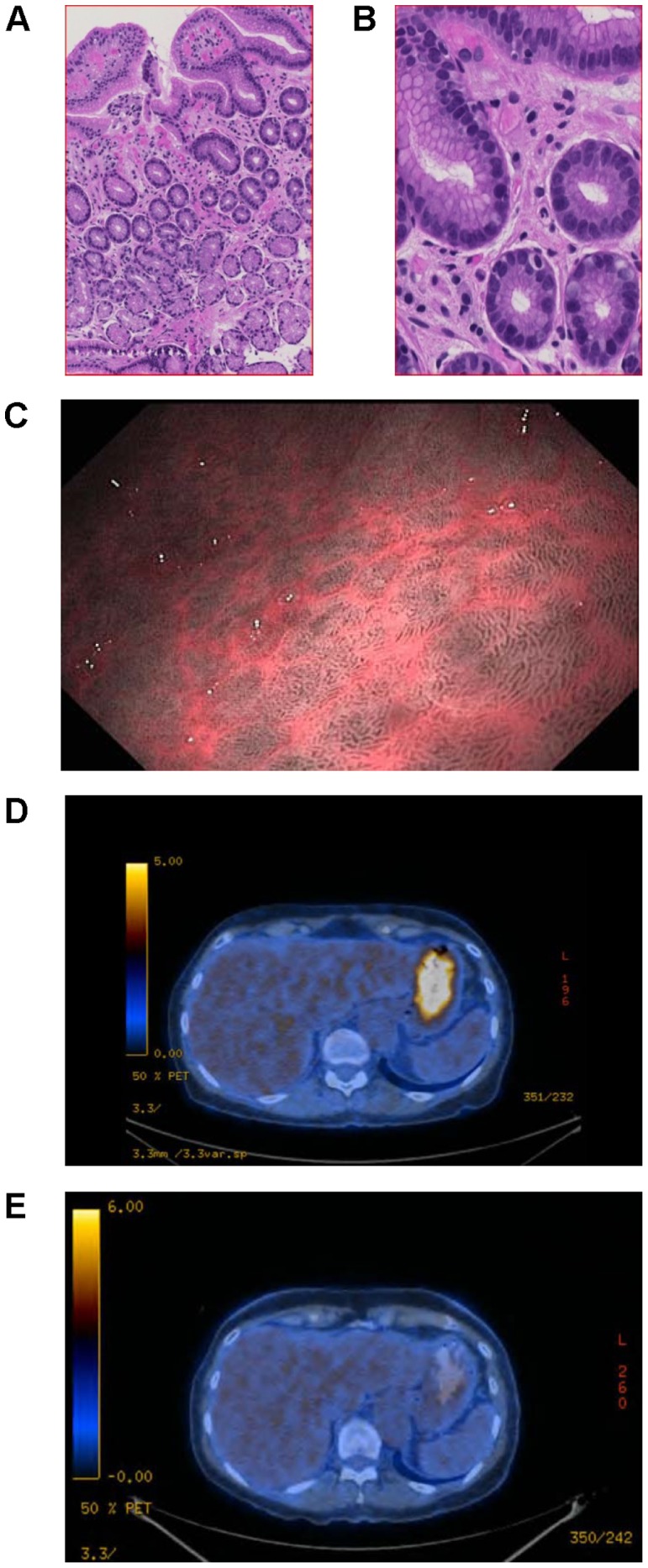
Ipilimumab-induced ischemic gastritis. Hematoxillin eosin staining showed edematous hypervascularized lamina propria mucosae, foveolar hyperplasia and regenerative basal crypts at 10× magnification (A) and 50× magnification (B). Endoscopic narrow band imaging (NBI) showed signs of reactive chronic inflammation of the gastric corpus mucosa with prominent vascular pattern consistent with an ischemic gastritis (C). Positron emission tomography (PET) scan illustrated high level tracer uptake in the gastric wall consistent with inflammation (D) and its spontaneous resolution after four months with a remaining thickening of the gastric wall (E).

A 72-year old female received ipilimumab (3 mg/kg body weight) due to progressive metastatic melanoma affecting lymph nodes, subcutaneous tissue and the gastrointestinal tract (cecum and jejunum without signs for passage disorders). Shortly after the first infusion, the patient underwent a complete surgical resection of all metastases with histological confirmation of the excised lesions. After the third infusion, the patient developed generalized pruritus including eyes and genital mucosa, which responded to antihistamines. Staging after the fourth treatment showed no metastases but a new strong diffuse fluordesoxyglucose (FDG)-enhancement in the gastric wall (most intense in the gastric corpus). Since the patient was asymptomatic with normal S100 values, radiologic follow-up without further action was advised. The next positron emission tomography (PET)-CT scan demonstrated no enhancement in the stomach mucosa. However, three months later, a strong FDG-enhancement in the corpus of the stomach was detected again, suggesting the presence of gastritis without further evidence of metastases. Radiological findings and an ongoing anemia eventually causing dyspnea, prompted a gastroscopy. Biopsy showed an ischemic gastritis compatible with the endoscopic findings (Figure 2). No helicobacter pylori (HP) or metaplasia were detected. Symptoms spontaneously resolved and the last PET-CT scan detected no enhancement of the gastric wall.

### Hepatitis

Hepatotoxicity is reported in 3–9% of ipilimumab patients and usually manifests as an asymptomatic increase of transaminases and bilirubin. Hepatitis has been reported in up to 0.8% of patients in the first [4] and up to 1.6% in the second phase III study [5]. Importantly, this AE can be life-threatening since one patient with fatal liver failure has been reported [4]. Thus, high dose steroids are recommended in case of grade 3/4 hepatotoxicity. In our study, 11 cases of liver-related irAEs were reported (Table 2, Table 3 patient 12; Table 4 last patient).

**Table 4:** Ipilimumab-induced miscellaneous reactions.

side effect	onset (weeksafter start ofipilimumab)	treatment^a^ ofside effect	outcome ofside effect	age(years)	gender	primarytumor	stageb,c	previoussystemictherapies	metestases*before*ipilimumab	remission*after*ipilimumab	clinicalresponse
tumor mass liquefication*	9	antibiotics, surgery	fatal	66	M	skin	IV	IFN-α, limb perfusion^f^ DTIC,temozolomide+vindesine+ cisplatin,fotemustine	LN, skin,intraabdominal,bone	LN, skin,intraabdomial	PR
grade IV anaphylactoid reaction	3	steroids anthistamines	resolved	54	M	skin	adjuvant	n/a	n/a	n/a	SD
swollen and numb hand	21	n/a	resolved	36	F	skin	adjuvant	n/a	n/a	n/a	SD
conjunctivitis	12	sodium hyaluronate eye gel	resolved	57	M	skin	IV	DTIC, sorafenib	LN, lung, adrenal glands, soft tissue, liver	lung	MR
non-septic arthritis	11	local steroids	resolved	41	F	skin	adjuvant	n/a	n/a	n/a	SD
non-septic arthritis	19	steroids	resolved	32	M	skin	adjuvant	n/a	n/a	n/a	SD
purulent sinusitis, VZV-infection	6^d^, 16^d^, 13^e^	antibiotics, valacyclovir	resolved	43	F	unkown primary	IV	sorafenib, temozolomide, fotemustine,	lung, liver spleen, brain	none	SD
sinusitis	14	antibiotics	resolved	38	M	skin	adjuvant	n/a	n/a	n/a	SD
sinusitis	3	antibiotics	resolved	53	M	skin	adjuvant	n/a	n/a	n/a	SD
myocardial fibrosis, portal livernecrosis with granulocytic infiltrates	16	n/a	fatal	61	F	skin	IV	vaccination^g^, IFN-α, vaccination^h^	skin, LN, lung, brain, epicardial, liver	n/a	PD

* case is detailed in the result section.

a listed treatments are systemic treatments unless otherwise specified.

b tumor-free high-risk stage III melanoma (AJCC 2009); adjuvant administration of ipilimumab melanoma.

c stage IV metastatic disease (AJCC 2009).

d sinusitis.

e VZV-infection.

f limb perfusion with melphalan.

g MAGE-A3 vaccination by GSK; NCT 00796445.

h PRAME; vaccination with GSK2302025A.

M indicates male; F, female; LN, lymph nodes; IFN-α, interferon-α; DTIC, dacarbazine; VZV, varicella-zoster virus; SD, stable disease; MR, mixed response; PD, progression of disease.

#### Patient 9– Fatal autoimmune hepatitis and nephritis

A 71-year old man with stable stage IV B-cell non-Hodgkin lymphoma suffered from metastatic melanoma. After the second ipilimumab treatment, the patient presented in a reduced general condition and with massive increase of liver transaminases and creatinine. Systemic steroids induced a slight improvement in liver transaminases within 24 hours. However, creatinine levels further increased and the patient required dialysis. In addition, his neurologic condition rapidly deteriorated with reduced responses to his environment and reduced respiration (spontaneous oxygenation below 60%). An inflammatory-induced capillary leak syndrome completely abolished renal function. Despite full symptomatic supportive treatment in the intensive care unit the patient died three days after admittance. Autopsy showed necrotic metastases and septal infiltration of the liver with CD3^+^ lymphocytes. This finding supports an ipilimumab-induced reaction and is less likely induced by lymphoma progression.

Unfortunately, this hepatotoxicity described above was steroid-refractory and resulted in fatal outcome. Importantly, successful treatment of a fulminant hepatitis refractory to treatment with steroids and mycophenolate mofetil with antithymocyte globulin (1.5 mg/kg for four times) has been reported [22].

### Endocrine System

IrAEs affecting the endrocrine system include euthyroid Graves ophthalmopathy [23] and thyroid dysfunction with both hypo- and hyperthyroidism, which can manifest as thyroiditis [24], [25]. Hypophysitis is a rare, yet serious complication of ipilimumab treatment. Incidence varies between 1.8% and 17% of patients at the 1–3 and 10 mg/kg doses, respectively [26], [27]. Symptoms include loss of libido, fatigue, headache, memory difficulties, dizziness, vision changes and constipation. When suspected, a complete work-up including serum potassium, sodium, morning cortisol, luteinizing hormone (LH), follicle stimulating hormone (FSH), testosterone, insulin-like growth factor (IGF)-1 and free thyroxine (fT4), as well as a brain magnetic resonance imaging (MRI) – also to exclude brain metastases - is necessary. Pituitary enlargement can precede clinical or laboratory evidence of an autoimmune-mediated hypophysitis. In contrast, hypophysitis cannot be ruled out by normal MRI findings. Upon diagnosis, prompt steroid therapy and regular follow-up with blood tests (serum potassium, sodium, testosterone (in men) and fT4) are indispensable. Cortisol measurements are not informative if steroid treatment is ongoing.

A newly diagnosed hyponaetremia or a secondary amenorrhoe seen in a premenopausal patient (patient 3, Table 3) are suspicious for newly developed corticotropin deficiency due to pituitary deficiency, whereas normal menses exclude gonadotropic deficiency in premenopausal women. Therapy consists of hormone replacement. Within this study, 14 patients with endocrinological AEs are reported including 12 cases of hypophysitis (Table 3).

#### Patient 10– Hypophysitis

A 74-year old male with no history of brain metastases received ipilimumab (3 mg/kg bodyweight) due to progressive metastatic disease affecting both adrenal glands. Shortly after the second treatment, he showed progressive ataxia and aphasia. MRI ruled out newly developed brain metastases and hypophysitis was suspected. Except for a slightly decreased testosterone, no hormonal changes were observed. Oral steroids (dexamethasone) were started and tapered. After two weeks, a decrease in thyroid hormones and testosterone was noted. Due to steroid therapy, the likewise decreased cortisol levels could not be used for interpretation. A second MRI demonstrated improvement of the morphological changes. Thyroid-hormone substitution was initiated and steroids tapered. After discontinuing steroid treatment, progressive ataxia and aphasia developed again. At this time, laboratory findings revealed hyponatraemia (serum sodium 125 mmol/l, normal range 135–145 mmol/l), and reduced levels of cortisol (2.6 µg/dl, normal range 5–25 µg/dl), testosterone (0.5 ng/ml, normal range 2.8–8.5 ng/ml) and dehydroepiandosterone (DHEAS) (<150 ng/ml, normal range 800–5000 ng/ml). Adrenocorticotropic hormone (ACTH), LH, prolactin, free triiodothyronine (fT3) and fT4 (under treatment with thyroid hormones) were normal. A third MRI showed similar results to the second MRI scan, but pituitary impairment persisted. Under i.v. steroids (dexamethasone 4×4 mg) ataxia and aphasia subsided and serum sodium levels normalized. In conclusion, the observed hyponatraemia appeared due to an acute Addison crisis after steroid withdrawal because of persisting corticotropin deficiency. Although the clinical course was further complicated by a steroid-induced psychosis and a bacterial infection of unknown origin, the patient is now free of symptoms under hormonal substitution with thyroid hormones, hydrocortisone and testosterone.

#### Patient 11 - Hypophysitis with brain edema

A 67-year old man with multiple melanoma metastases received stereotactic irradiation for a single parietooccipital brain metastasis (4 mm diameter) and developed a new cerebellar metastasis. Four consecutive treatments with ipilimumab (3 mg/kg body weight) were well tolerated apart from a slight decrease in thyroid-stimulating hormone (TSH) (0.3 mU/l; normal range: 0.4–4.9 mU/l) with normal fT4 before the third treatment. After the third treatment the cerebellar metastasis was stereotactically irradiated. Fourteen weeks after initiation of ipilimumab, the patient presented with acute onset of nausea, dizziness and ataxia. On examination, a blurred speech was noticed, prolactin was elevated with 30.7 ng/ml (normal range 3.5–19.5 ng/ml), cortisol and TSH were decreased with <1.0 µg/dl (normal range 4.3–22.4 µg/dl) and 0.27 mU/l (normal range 0.4–4.9 mU/l), respectively. CT scan showed the previously described irradiated metastases with adjacent edema as well as an increase in volume of the pituitary gland with signs of brain edema. Under i.v. steroid treatment (dexamethasone 16 mg/day) symptoms ameliorated.

In agreement with previous reports, 4 out of 12 hypophysitis patients require ongoing hydrocortisone replacement and in some cases additionally thyroid hormones and testosterone/estrogen [28], [29].

### Nervous System

Ipilimumab-associated neurological symptoms are rare, but may be life-threatening. Common symptoms include headache, dizziness, lethargy, and asthenia. Rarely, patients present with cranial neuropathy and optic nerve ischemia, ataxia, tremor, myoclonia, dysarthria and peripheral neuropathy. Importantly, Guillian-Barré syndrome was described twice [30] with fatal outcome in one patient [4]. In addition, meningo-radiculo-neuritis [31], enteric neuropathy [32] and cerebral edema with convulsions [33] have been reported.

Six out of eleven rare neurological AEs reported in this study required immunosuppression (Table 5, last patient Table 6). In general, steroid treatment was effective although one patient experienced a therapy-refractory neuropathy and eventually died despite additional treatment with intravenous immunoglobulin (IVIG).

**Table 5:** Ipilimumab-induced reactions of the nervous system.

side effect	onset (weeksafter start ofipilimumab)	treatment^a^ ofside effect^a^	outcome ofside effect	age(years)	gender	primarytumor	stageb,c	previoussystemictherapies	metastases*before*ipilimumab	remission*after*ipilimumab	clinicalresponse
Tolosa-Hunt-Syndrom*	18	steroids	ongoing	M	65	skin	IV	IFN-α, TKI (RAF265), DTIC	LN, soft, tissue, GIT	PD	PR
granulomatous inflammation ofthe central nervous system*	10	steroids, fotemustine	resolved	M	50	skin	adjuvant	none	brain, LN	n/a	PD
aseptic meningitis*	4	steroids, acyclovir, antibiotics	resolved	F	52	skin	IV	DTIC	LN, soft tissue, liver, bones	none	PD
dysgeusia	8	n/a	ongoing	M	66	skin	adjuvant	n/a	n/a	n/a	SD
dysgeusia	35	n/a	ongoing	F	44	skin	adjuvant	n/a	n/a	n/a	SD
facial nerve paralysis	7	steroids (0.65 mg/kg)	resolved	M	61	mucosal	IV	IFN-α	lungs	none	PD
neuralgiform pain	31	pregabalin	ongoing	M	53	skin	adjuvant	n/a	n/a	n/a	SD
therapy refractory neuropathy	2	steroids, venlaflaxin, hydromorphone, IVIG, pregabalin	ongoing (till death)	M	50	skin	IV	IFN-α	LN, skin, liver, brain, kidney	none	PD
tinnitus, acute hearing loss, chills/shivering, diarrhea, generalized pruritus	1	betahistin, loperamide	resolved	M	50	skin	IV	IFN-α, DTIC	LN, soft tissue, brain	LN, soft tissue, brain	PR
generalized epileptic seizure	2	steroids, levetiracetam	resolved	M	72	unknown primary	IV	paclitaxel, vemurafenib	lung, skin, brain	none	PD

* case is detailed in the result section.

a listed treatments are systemic treatments unless otherwise specified.

b tumor-free high-risk stage III melanoma (AJCC 2009); adjuvant administration of ipilimumab.

c stage IV metastatic disease (AJCC 2009).

M indicates male; F, female; LN, lymph nodes; IFN-α, interferon-α; DTIC, dacarbazine; TKI, tyrosine kinase inhibitor RAF265; GIT, gastrointestinal tract; PR, partial response; SD, stable disease; PD, progressive disease.

**Table 6:** Ipilimumab-induced reactions of the respiratory tract and renal system.

side effect	onset (weeksafter start ofipilimumab)	treatment^a^ ofside effect	outcome ofside effect	age(years)	gender	primarytumor	stageb,c	previoussystemictherapies	metastases*before*ipilimumab	remission*after*ipilimumab	clinicalresponse
barky rhinitis	11	steroids	resolved	F	49	skin	IV	bevacizumab, temozolomide, DTIC, eldesine, platinol, paclitaxel, sorafenib	lung, liver, soft tissue, pancreas, LN, bones, skin, GIT	liver, LN	MR
alveolitis	3	steroids	resolved	M	59	unknown primary	IV	temozolomide	brain, lung	none	PD
persistent bronchitis(>3 months in summer)	17	antibiotics	resolved	M	38	skin	adjuvant	n/a	n/a	n/a	SD
dyspnea	14	inhalative steroids	resolved	F	44	skin	adjuvant	n/a	n/a	n/a	SD
intermittent dyspnea	39	steroids	resolved	M	64	skin	adjuvant	n/a	n/a	n/a	SD
cough, dyspnea, arthritis, myalgia, diarrhea, sweating, papular exanthema	1	acetylcysteine	resolved	M	48	skin	IV	IFN-α, DVP	LN, lung, liver, bone	LN, liver	PR
acute renal failure, interstitial nephritis, atypical pneumonia	6^e^, 10^f^	steroids, antibiotics	resolved	F	72	unknown primary	IV	DTIC, vaccination (PRAME)^d^	skin, LN	LN	PR
acute renal failure, atypical pneumonia, iridocyclitis/keratitis, deafness	8g, 10^h^	steroids (1 mg/kg )	resolved^i^, permanent changes^j^	F	53	mucosal	IV	IFN-α, DTIC, sorafenib, carboplatin+paclitaxel, fotemustine	kidney, skin, paracolic area, spinal cord	kidney, skin, paracolic area, spinal cord	PR

* case is detailed in the result section.

a listed treatments are systemic treatments unless otherwise specified.

b tumor-free high-risk stage III melanoma (AJCC 2009); adjuvant administration of ipilimumab.

c stage IV metastatic disease (AJCC 2009).

d PRAME study; vaccination with GSK2302025A.

e atypical pneumonia.

f acute renal failure.

g renal failure/atypical pneumonia.

h iridocyclitis/keratitis, deafness.

I renal failure/atypical pneumonia/iridocyclitis/keratitis.

j deafness.

M indicates male; F, female; LN, lymph nodes; IFN-α, interferon-α; DTIC, dacarbazine; DVP; polychemotherapy with dacarbazine/vindesine/paclitaxel; GIT, gastrointestinal tract; PR, partial response; SD, stable disease; MR, mixed response; PD, progressive disease.

#### Patient 12– Granulomatous inflammation of the central nervous system (CNS)

A 50-year old man with stage IIIA melanoma experienced transient chills without fever eight weeks after initiation of ipilimumab. In addition, two weeks later right-sided facial paresthesia and muscle weakness in both legs were observed. Paresthesia in his face deteriorated and extended to the whole face. Additionally, singultus and nausea appeared and persisted. Twelve weeks after start of ipilimumab, his neurological condition suddenly worsened resulting in sensoric ataxia and a disabling progressive paraparesis affecting both legs. All examinations for bacterial, fungal and viral causes were negative. No melanoma cells could be detected by lumbar puncture, and a marked lymphocytic pleocytosis was seen. Blood CD4/CD8 ratio was markedly increased at 4.3 (normal range 1.1–3.0) with an increase of absolute CD4-lymphocytes (1.8/nl; normal range 0.5–1.2/nl). The MRI showed an enhancement in both trigeminal nerves and, additionally, three parenchymal lesions with no indication for meningiosis carcinomatosa or pituitary enlargement. Because of suspected ipilimumab-induced granulomatous disease high dose steroids were initiated and quickly improved symptoms. Brain lesions disappeared in a subsequent MRI and symptoms completely resolved.

#### Patient 13– Tolosa-Hunt-Syndrome

A 65-year old male patient with primary skin melanoma without brain metastases or thyroid disease received ipilimumab (3 mg/kg body weight) due to progressive metastatic melanoma. Eighteen weeks after the first dose of ipilimumab, the patient presented with acute onset of strong pain above his right eye that radiated to the paranasal sinus, epiphora and double vision. In addition, dizziness and nausea was reported. The ophthalmologist recorded a mydriasis of the right pupil and a ptosis with paresis of the oculomotorius nerve leading to limited mobility and inability to completely open the right eye, compatible with a Tolosa-Hunt-Syndrome. Brain MRI revealed a markedly prominent neural sheath of the right optic nerve, no alteration in the cavernous sinus and no metastases. Except for a stable anemia and an increased CRP (54 mg/l, normal range <5 mg/l), laboratory values were within normal range with normal TSH and negative thyroid autoantibodies. Interestingly, an unclear FDG-enhancement in the left thyroidal lobe had been previously detected by FDG-PET/CT. High-dose steroids (initially i.v. methylprednisolone and subsequently oral dexamethasone) and local radiotherapy (10×3 Gray) was simultaneously started. This treatment combination markedly and promptly reduced the ocular pain and paresis but showed only little impact on the vision disturbances. Staging showed progressive disease with new liver metastases.

#### Patient 14– Aseptic meningitis

A 52-year old female with melanoma metastases affecting lymph nodes, soft tissue, liver, and bones (sacral vertebra, 2 ribs and temporal bone) presented with nausea, vomiting, chills and rash, three weeks after the first ipilimumab infusion. Therapy included rehydration, antiemetics, novaminsulfon and topical steroids. One week later, she presented with agitation, disorientation, aggressive behaviour and the inability to make contact with other people. Since she was screaming and physically attacking the physicians, a blood draw required four people holding the patient in addition to sedation with esketamin (50 mg) and midazolam (15 mg). Body temperatures had risen to 39°C but no signs of meningism were observed. Treatment with i.v. ceftriaxon, ampicillin as well as acyclovir and dexamethasone was initiated. Liquor analyses revealed mostly lymphomonocytic cells (mainly CD3-positive lymphocytes) and excluded a meningiosis neoplastica or herpes simplex infection. Brain MRI showed four new brain metastases (<1 cm in diameter each) and an accentuation of the temporal bone metastasis. After electroencephalography (EEG), transcranial ultrasound, and repeated liquor examinations, an aseptic ipilimumab-induced meningitis was suspected. Ipilimumab treatment was permanently discontinued and symptoms resolved completely. Unfortunately, the patient demonstrated disease progression despite subsequent gamma knife and fotemustine treatment.

Physicians should be aware that neurologic symptoms can exacerbate unexpectedly at any time. The patient described above would have died of meningitis if no relatives would have been around. Importantly, it has been reported that prior therapy with neurotoxic agents may increase the risk of neurological-related adverse reactions [30], however, these findings are not confirmed in our study population.

### Respiratory Tract and Renal System

Reported ipilimumab-induced respiratory tract-related adverse reactions include a life-threatening pneumonitis after allogeneic hematopoietic cell transplantation [34], fatal acute respiratory distress syndrome (product monography), pulmonary granulomatosis [35] and sarcoidosis [9], [36], [37]. Observed rare respiratory-tract irAEs, included barky rhinitis, alveolitis and atypical pneumonia (Table 6). Ipilimumab-induced respiratory tract-related irAEs have significant clinical implications, since they may be life-threatening. In addition, radiological signs of sarcoidosis can be confused with pulmonary metastases [38] resulting in inadequate change of therapy.

Adverse reactions affecting the renal tract are rare [7]. In this study two cases of acute renal failure were reported (Table 6). Both responded well to steroid treatment and resolved without sequelae.

### Miscellaneous

It remains unclear why different targets are affected during ipilimumab treatment. In the literature, hemophilia A [39] and autoimmune polymyositis [40] have been reported.

**Figure 3:**
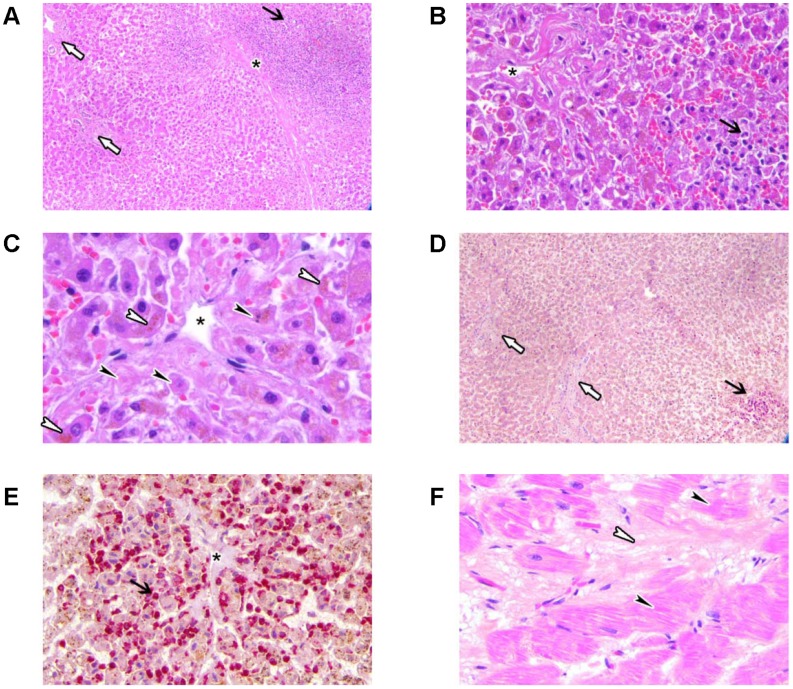
Ipilimumab-induced myocardial fibrosis in conjunction with hepatotoxicity. Hematoxillin eosin staining at 50× magnification (A), 200× magnification (B) and 400× magnification (C) and chloracetate esterase staining at 50× magnification (D), 200× magnification (E) and 400× magnification (F) revealed neutrophilic granulocytes (black arrow) mostly around the central vein (asterisk). Portal fields were almost normal (white arrows). Some necrotic hepatocytes (black arrow heads panel C) and cholestasis of hepatocytes (white arrow heads panel C) indicating liver insufficiency, were detected pericentrally. Slightly elevated myocardial fibrosis (white arrow heads panel F) surrounded by structural changes of cardiomyocytes were detected (black arrow heads panel F).

In our study, eleven patients presented with AEs not related to skin, liver, endocrine system, respiratory tract, kidney, or nervous system (Table 4). Interestingly, one patient showed myocardial fibrosis in conjunction with hepatitis (Figure 3). Importantly, one patient experienced an anaphylactoid reaction with flush, diffuse muscle contractions, chest tightness, hypertension, tachycardia and tachypnoe after infusion of 20% of the total ipilimumab volume of the second infusion. Symptoms resolved upon treatment with i.v. steroids and antihistamines. To the best of our knowledge, this is the first reported grade IV anaphylactoid reaction to ipilimumab.

#### Patient 15 - Tumor mass liquefication

**Figure 4:**
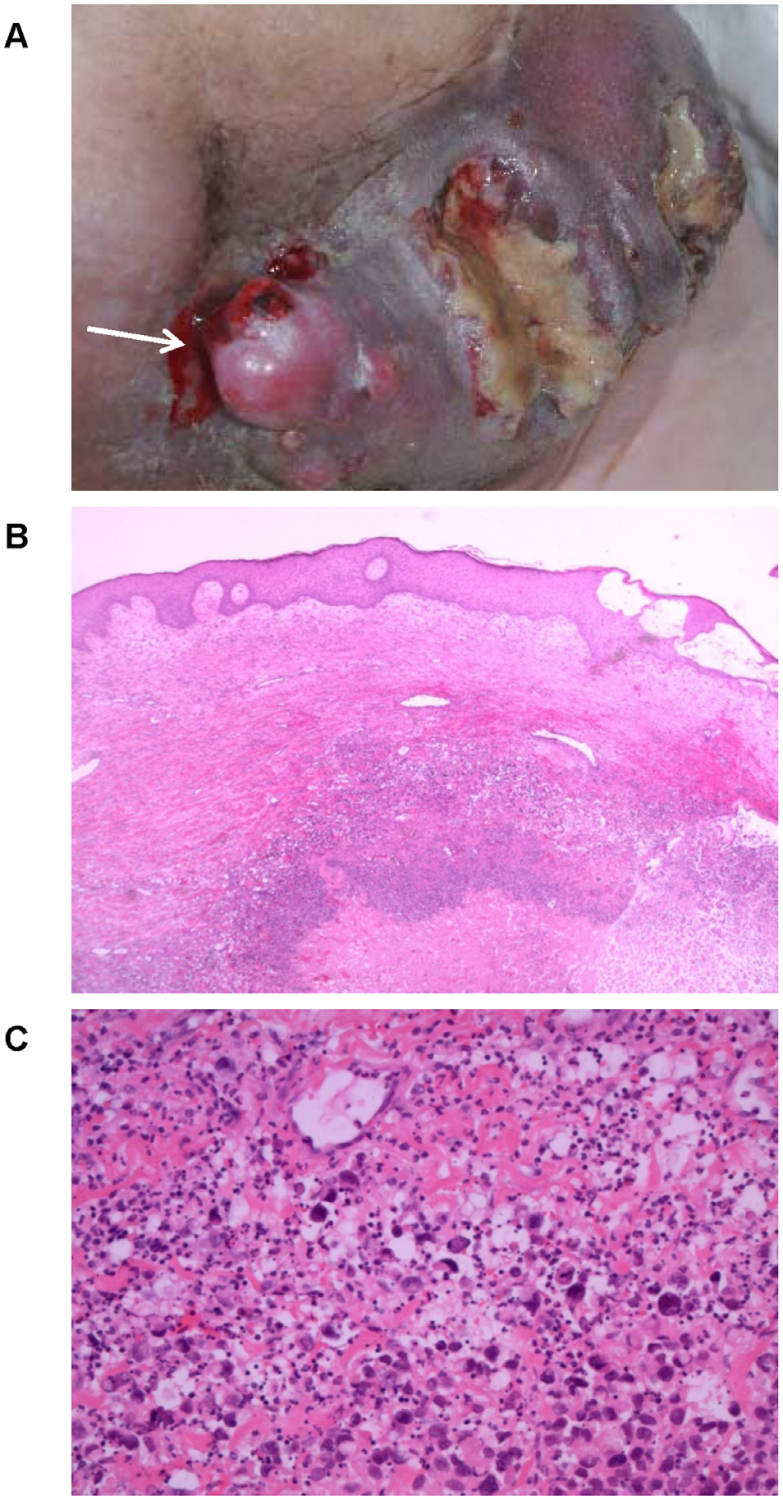
Ipilimumab-induced tumor mass liquefication. Ulcerated, partially liquefied tumor mass inguinal left (A). Histologic examination confirmed an abundance of necrotic tumor cells with leukocytic infiltration and residual highly pleomorphic tumour cells, haemorrhage and fibrosis (images in hematoxillin eosin staining, magnification 200×; B+C).

A 66-year old male patient presented with progressive left-sided iliac lymph node metastases and bulky tumor growth on the left-sided abdomen, the groin and left thigh despite lymph node dissection, hyperthermal limb perfusion, dacarbazine monochemotherapy, polychemotherapy with temozolomide, vindesine and cisplatin and fotemustine. In addition, arterial and venous compression, infiltration and thrombosis led to a massive increase of the pre-existing lymph edema of the left leg. An attempt of brachytherapy failed. Upon the second treatment with ipilimumab, the patient reported a considerable reduction of the tumor mass and lymph edema. At this time, the tumor bulk on the left-sided abdomen showed two ulcerations with minimal foetid secretion (Figure 4A). Four weeks later, tumor infiltration and lymph edema of the left leg were nearly resolved. Both ulcerations were still present and sore. Abdominal examination revealed a ballottement and a highly inflammatory induration of the entire lower abdominal wall. No hyperphosphatemia, hyperkalemia, hyperuricemia or hypocalcemia were observed. An ultrasound-guided tumor incision on the right side of the abdomen released approximately 400 ml of a liquefied, partially necrotic and putrefied tumor bulk. Further CT-controlled drainage of the abdominal mass did not result in any improvement and the patient unfortunately died shortly after the third treatment despite antiseptic treatment including broad spectrum antibiotics, presumably as a consequence of septicaemia.

This case illustrates ipilimumab’s potential to induce strong antitumor immune responses with rapid and sustained tumor destruction (Figure 4B–C). Tumor destruction releases antigens, including non-self antigens as well as endogenous danger signals, which trigger inflammation, cytokine release and the infiltration of immune cells in the tumor-affected organs. Extended inflammation, tumor destruction and necrosis may induce multi-organ failure similar to a tumor lysis syndrome.

## Discussion

This study summarizes unexpected and rare ipilimumab-induced AEs. To our knowledge, we report for the first time on (i) rare skin reactions, including a DRESS, a photosensitivity reaction and skin toxicity in a previously radiated area, (ii) a case of ischemic gastritis, (iii) rare neurological reactions, including granulomatous CNS inflammation, a Tolosa-Hunt-Syndrome and aseptic meningitis and (iv) a case of tumor mass liquefication with fatal outcome. Furthermore, we report on three intestinal perforations of which one was masked by ongoing steroid therapy, one occurred despite therapy with steroids and infliximab and one occurred in the small intestine outside the endoscopic examination range. In addition, different courses of ipilimumab-induced hypophysitis are described. Time course of specific side effects differed with e.g., much earlier onset of hepatitis than previously reported [20].

Since ipilimumab-induced irAEs can virtually affect any organ system physicians have to consider all symptoms as potentially ipilimumab-associated. In turn, patients have to be instructed to report all symptoms even if deemed unrelated. Several centers experienced difficulties in patient compliance with reporting AEs. For example, patients with severe diarrhea mostly reported symptoms several days after onset, which in one case resulted in a colonic perforation. As compliance of patients is limited when they fear their treatment will be stopped due to AEs, the fact that treatment efficacy is not abrogated despite steroid treatment should be stressed [41], [42]. In the case of ipilimumab-induced irAEs prompt steroid treatment reduces intensity and duration of symptoms [43]. Even conditions that classically do not respond to steroid treatment, like Guillain-Barré syndrome [30] or hypophysitis, readily respond if ipilimumab-induced. Among all 120 evaluated patients in this study, side effects resolved in 82 cases. In 22 patients, side effects are ongoing and in 13 patients permanent changes remained. Unfortunately, three ipilimumab-induced fatalities occurred despite treatment.

Side effects caused by other drugs have to be differentiated from ipilimumab-induced irAEs as seen in patient 10 with psychological symptoms, first due to an ipilimumab-induced hypophysitis and later due to steroid treatment. Similarly, steroid-induced myopathy in a colitis patient can resemble an irAE. Thus, in order to avoid steroid-induced side effects in prolonged autoimmune reactions, a switch to other immunosuppressants, like etanercept, infliximab, or mycophenolat mofetil may be advisable. Furthermore, skin- and gut-related adverse reactions may reflect immune activation in response to signals from commensal organisms [44]. However, in the skin immunoactivation through commensal microflora seems less likely, since most ipilimumab-induced skin reactions morphologically show exanthema, rather than eczema. In patients with colitis, detailed studies addressing antigen-specificity of ipilimumab-induced immune reactions are needed to distinguish autoimmunity from an enhanced reaction to resident flora.

Frequency and severity of irAEs seem to be dose-dependent [12], [13]. However, immunotherapeutics in general do not show linear dose response curves since induction of immunity depends on the host’s immune system. Limited data exists to predict response or identify patients who are likely to develop severe irAEs. CTLA-4 polymorphism may play a role [45] although clear evidence is still pending [46]. Importantly, genetic predisposition for the development of autoantibodies is postulated since mice expressing specific CTLA-4 isoforms developed spontaneous autoimmunity, including elevated production of autoantibodies [47]. In addition, anti-CTLA-4 antibodies have been shown to induce autoantibodies in mice [48] and CTLA-4 specific autoantibodies have been found in sera from patients suffering from various autoimmune diseases, including systemic lupus erythematosus, rheumatoid arthritis, systemic sclerosis, Behçet’s disease and Sjögren’s syndrome [49]. Although autoantibodies may be generated in vivo by an antigen-dependent mechanism and are postulated to modulate immune responses by interfering with CTLA-4 on T cells, it remains unclear whether CTLA-4 specific autoantibodies contribute to or protect against autoimmune reactions. Future studies in patients undergoing ipilimumab-treatment will be necessary to elucidate this question.

Interestingly, several large studies reported increased efficacy in patients affected by irAEs [21], [42], [50]–[52] with responses in 26% of patients experiencing any irAE compared to 2% in patients, who did not experience any irAE [42]. There also was a ‘severity-response-effect’ with response rates of 22% and 28%, in patients with grade 1/2 and grade 3/4 adverse reactions, respectively [42]. Nevertheless, clinical responses are also seen in patients treated with ipilimumab without any irAE [50]. Furthermore, it is unclear whether four infusions as currently approved for treatment are necessary to induce tumor response. In patients where treatment was interrupted due to irAEs, clinical benefit was already observed after 1–3 ipilimumab infusions. This suggests that in a subgroup of patients fewer infusions might be sufficient to boost pre-existing anti-tumor immunity. A reduced number of infusions - if proven effective - could reduce costs as well as associated irAEs.

In our study, tumor regression was observed in 30.9% (21 out of 69) and tumor control in 61.8% (42 out of 69) of stage IV patients. No association was observed between organ system affected by the side effect and organ system that responded to therapy. Since ipilimumab can only augment existing T-cell responses, a previous tumor-specific immune response is indispensable [53] and could be induced by prior antigen-specific immunotherapy.

## Supporting Information

Table S1 **Participating centers, number of patients treated, dosages administered, and treatment settings.**
(DOC)
