# Supplementary material for: The Price of Tumor Control: An Analysis of Rare Side Effects of Anti-CTLA-4 Therapy in Metastatic Melanoma from the Ipilimumab Network
Source: PLoS One. 2013 Jan 14;8(1):e53745. doi: 10.1371/journal.pone.0053745 (PMC3544906; doi:10.1371/journal.pone.0053745)
Supplement: Table S1 — Participating centers, number of patients treated, dosages administered, and treatment settings. (DOC) [file pone.0053745.s001.doc]

**Table S1 Participating centers, number of patients treated, dosages administered, and treatment settings**

| **center** | **nr. of patients** | **dosages administered (mg/kg body weight)** | **treatment settings** |
| --- | --- | --- | --- |
| University Hospital Charité Berlin, Germany | 34 | 3 mg/kg | 1,2,3 |
| Elbe Hospital Buxtehude, Germany | 39 | 3 mg/kg | 1,3,4,5 |
| Cantonal Hospital Chur, Switzerland | 6 | 3 mg/kg | 3,5 |
| University Hospital Essen, Germany | 66 | 3 mg/kg , 10 mg/kg | 1,2,3,4,5 |
| University Hospital Erlangen, Germany | 11 | 3 mg/kg | 1,3,5 |
| University Hospital Frankfurt, Germany | 26 | 3 mg/kg | 3, 5 |
| University Hospital Graz, Austria | 20 | 3 mg/kg | 3,5 |
| University Hospital Hannover, Germany | 41 | 3 mg/kg, 10 mg/kg | 3,4,5 |
| Hospital Hornheide, Germany | 6 | 3 mg/kg | 1 |
| University Hospital Kiel, Germany | 60 | 0,3 mg/kg, 3 mg/kg, 10 mg/kg | 1,3,5,6,7,8 |
| University Hospital Magdeburg, Germany | 8 | 3 mg/kg | 3,5 |
| University Hospital Mainz, Germany | 50 | 3 mg/kg | 1,3,4,5 |
| University Hospital Munich LMU, Germany | 19 | 3 mg/kg | 1,3,5 |
| University Hospital Munich, TU, Germany | 40 | 3 mg/kg , 10 mg/kg | 1,3,6,9 |
| Institute Gustave Roussy, Paris, France | 156 | 3 mg/kg , 10 mg/kg | 2,3,5,6,9,10 |
| Hospital Quedlinburg, Germany | 9 | 3 mg/kg | 1,3,5 |
| Cantonal Hospital St. Gallen, Switzerland | 13 | 3 mg/kg | 3,5 |
| University Hospital Tuebingen, Germany | 52 | 3 mg/kg | 1,2,3 |
| University Hospital Zurich, Switzerland | 96 | 3 mg/kg , 10 mg/kg | 2,3,5,9 |
|  |  |  |  |
| **total** | **752** |  |  |

(1) DeCOG-MM-PAL11 (NCT01355120): Multicenter, prospective phase II multibasket study in pretreated stage III-IV metastatic melanoma patients. Ipilimumab was administered according to the MDX010-20 protocol every 3 weeks at 3 mg/kg body weight.

(2) CA184-029; EORTC 18071 (NCT00636168): Randomized, double-blind phase III clinical trial comparing the adjuvant administration of ipilimumab with placebo after complete resection of high-risk stage III melanoma. Ipilimumab was administered every 3 weeks at 10 mg/kg body weight.

(3) Prescribed treatment after EMA approval. Ipilimumab was administered every 3 weeks at 3 mg/kg body weight.

(4) CA 184-169 (NCT01515189): Randomized double-blind phase III clinical trial of ipilimumab in previously treated, untreated unresectable or metastatic stage III/IV melanoma patients. Ipilimumab was administered every 3 weeks at 3 mg/kg or 10 mg/kg body weight.

(5) Compassionate Use/Extended Access Program (NCT00495066): Ipilimumab monotherapy in patients with previously treated, unresectable and/or metastatic stage III/IV melanoma. Ipilimumab was administered every 3 weeks at 3 mg/kg body weight.

(6) CA 184-024 (NCT00324155): Randomized, double-blind, multicenter phase III clinical trial comparing dacarbazine monotherapy with dacarbazine plus ipilimumab in patients with unresectable and/or metastatic stage III/IV melanoma. Ipilimumab was administered every 3 weeks at 10 mg/kg body weight.

(7) CA 184-022: Ipilimumab monotherapy in patients with previously treated unresectable stage III or IV melanoma. Ipilimumab was administered every 3 weeks at 0.3 mg/kg, 3 mg/kg or 10 mg/kg body weight then every 12 weeks for 48 weeks.

## (8) CA 184-025: a multicenter, open-label, phase II study of Ipilimumab (MDX-010) extended-treatment Monotherapy or follow-up for patients previously enrolled in Ipilimumab (MDX-010) protocols. Follow-up study with re-induction of ipilimumab at a dose of 10 mg/kg body weight.

(9) CA 184-002 (NCT00094653): Randomized, double-blind multicenter phase III clinical trial comparing ipilimumab monotherapy, ipilimumab in combination with a gp100 peptide vaccine and gp100 peptide vaccine monotherapy in HLA-A2*0201 positive, previously treated patients with unresectable stage III/IV metastatic melanoma. Ipilimumab was administered every 3 weeks at 3 mg/kg body weight.

(10) Mel-Ipi-Rx (NCT01557114): Dose-escalation phase I clinical trial of radiotherapy administered in combination with ipilimumab in patients with unresectable and/or metastatic stage III/IV melanoma. Ipilimumab was administered every 3 weeks at 10 mg/kg body weight.
